# Attempts to remodel the pathways of gemcitabine metabolism: Recent approaches to overcoming tumours with acquired chemoresistance

**DOI:** 10.20517/cdr.2020.39

**Published:** 2020-10-12

**Authors:** Yuriko Saiki, Shuto Hirota, Akira Horii

**Affiliations:** ^1^Department of Molecular Pathology, Tohoku University School of Medicine, Sendai, Miyagi 980-8575, Japan.; ^2^Office of Medical Education, Tohoku University School of Medicine, Sendai, Miyagi 980-8575, Japan.; ^3^Saka General Hospital, Shiogama, Miyagi 985-0024, Japan.

**Keywords:** Gemcitabine, chemoresistance, deoxycytidine kinase, human equilibrative nucleoside transporter 1, cytidine deaminase, ATP-binding cassette transporters, metabolism

## Abstract

Gemcitabine is a cytidine analogue frequently used in the treatment of various cancers. However, the development of chemoresistance limits its effectiveness. Gemcitabine resistance is regulated by various factors, including aberrant genetic and epigenetic controls, metabolism of gemcitabine, the microenvironment, epithelial-to-mesenchymal transition, and acquisition of cancer stem cell properties. In many situations, results using cell lines offer valuable lessons leading to the first steps of important findings. In this review, we mainly discuss the factors involved in gemcitabine metabolism in association with chemoresistance, including nucleoside transporters, deoxycytidine kinase, cytidine deaminase, and ATP-binding cassette transporters, and outline new perspectives for enhancing the efficacy of gemcitabine to overcome acquired chemoresistance.

## Introduction

**Figure 1:**
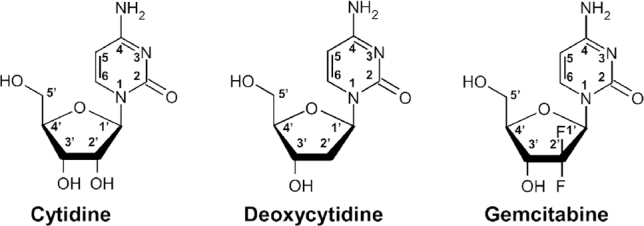
Structures of cytidine, deoxycytidine and gemcitabine

**Figure 2:**
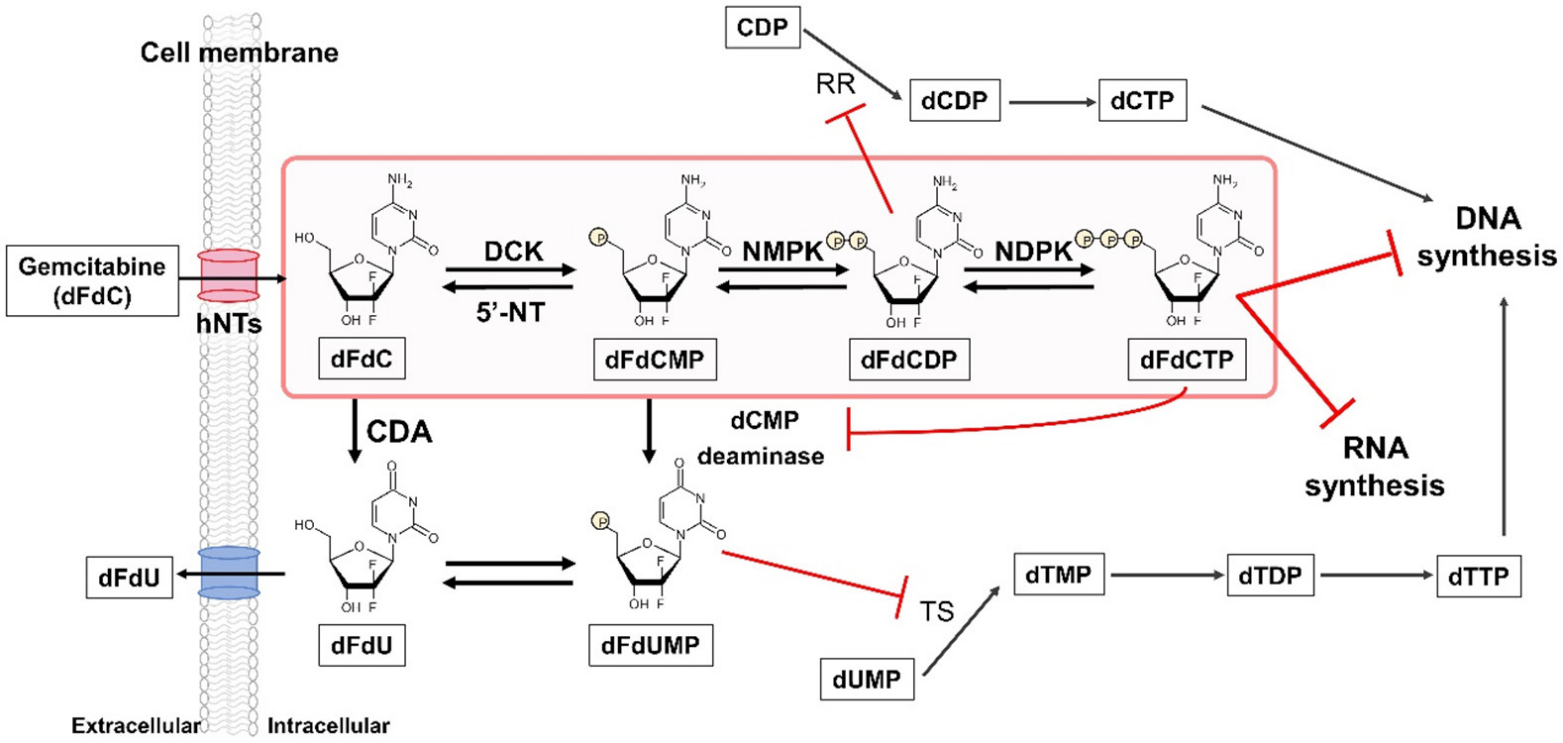
Metabolism and action of gemcitabine [difluoro 2’-deoxycytidine (dFdC)]. dFdC is transported into the cell through nucleoside transporters (hNTs), then stepwise phosphorylated by deoxycytidine kinase (DCK), nucleoside monophosphate kinase (NMPK), and nucleoside diphosphate kinase (NDPK), to form active triphosphate metabolite (dFdCTP). This molecule then inhibits DNA and RNA synthesis. Diphosphate metabolite (dFdCDP) inhibits ribonucleotide reductase (RR), an enzyme that catalyses the conversion of ribonucleotide (CDP) to deoxyribonucleotide (dCDP). The majority of dFdC is inactivated mainly by cytidine deaminase (CDA) mediated conversion to difluorodeoxyuridine (dFdU) and then excreted through the ABC transporter. Deamination of dFdCMP to dFdUMP by deoxycytidylate deaminase (dCMP deaminase) and subsequent dephosphorylation forms dFdU; this is another inactivation pathway of dFdC. dFdUMP inhibits thymidylate synthase (TS), resulting in the depletion of the dTMP pool. dFdCTP inhibits dCMP deaminase

Gemcitabine [2’,2’-difluoro-2’-deoxycytidine (dFdC)], was first described by Eli Lilly and Company in 1986^[1]^ and is the most important deoxycytidine nucleoside analogue with fluorine substituents at the 2’ position of the pentose ring [Figure 1]^[2]^. Its metabolic pathway is illustrated in Figure 2. This molecule is hydrophilic, and can be transported into cells by nucleoside transporters (hNTs), including both sodium-dependent concentrative nucleoside transporters (hCNTs) and sodium-independent equilibrative nucleoside transporters (hENTs). hCNTs mediate unidirectional transportation of nucleosides. hENT1 can uptake gemcitabine with high affinity but low capacity, whereas hENT2 can uptake gemcitabine with low affinity but high capacity. The intracellular uptake of gemcitabine is mainly mediated by hENT1 in cancer cells. In hepatocytes, the uptake of gemcitabine is mainly mediated by low affinity hENT2^[3,4]^.

Gemcitabine is a prodrug which requires intracellular phosphorylation for activation. Inside the cell, gemcitabine is phosphorylated to its monophosphate form (dFdCMP) by deoxycytidine kinase (DCK) and is then further phosphorylated to its diphosphate (dFdCDP) and then triphosphate forms (dFdCTP), as shown in Figure 2. The resulting dFdCTP is incorporated into DNA and then the DNA strand synthesis is terminated after incorporation of another nucleotide, hiding dFdCTP from DNA repair enzymes^[5]^. dFdCTP is also incorporated into RNA^[6,7]^, and sensitivity to gemcitabine is related to differences in RNA incorporation^[8]^. RNA incorporation of gemcitabine may play an important role in its activity. dFdCDP is an effective inhibitor of ribonucleoside-diphosphate reductase, an enzyme that transforms CDP into dCDP; this results in a decrease of the dCTP pool. Deamination of dFdCMP by dCMP-deaminase forms dFdUMP. Thymidylate synthase, which plays a key role in the synthesis of thymidine monophosphate (TMP)^[9]^, is another target for gemcitabine, via dFdUMP. The natural substrate of TS, 2’-deoxyuridine monophosphate (dUMP), resembles dFdUMP, and it inhibits TS resulting in a depletion of the TMP pool.

Evidence for the usefulness of gemcitabine as a potent anti-tumour reagent has been reported; it is used either alone, or in combination with other agents for patients with pancreatic ductal adenocarcinoma (PDAC)^[10]^ and several other human cancers, such as non-small cell lung cancer, breast cancer, ovarian cancer, and bladder cancer^[11]^ (approved by FDA). However, acquisition of chemoresistance against gemcitabine significantly limits its effectiveness. Chemoresistance can be divided into two categories, intrinsic and acquired, in the course of drug treatment^[12]^. Activities of drug transporters and metabolizing enzymes have been considered to be strongly involved in the chemoresistance to gemcitabine. Epithelial-to-mesenchymal transition (EMT) is not only related to a phenotypic change in the tumour cells; it also contributes to gemcitabine resistance^[13]^. Based on gene expression profiles of pancreatic cancer cell lines, gemcitabine-resistant cells contain many features consistent with EMT^[14]^. Exosomes have shown to be involved in gemcitabine resistance by delivering miRNAs. Exosomal miR-106b from cancer-associated fibroblasts^[15]^ and miR-210 from cancer stem cells^[16]^ both promote gemcitabine resistance. However, these areas are beyond the focus of this review, and we will discuss the challenges of remodelling the gemcitabine metabolizing pathway to overcome acquired chemoresistance against gemcitabine.

## Improvement of gemcitabine uptake

The membrane permeability of gemcitabine is poor in human cells. It is mediated by five distinct hNTs with different affinities; two equilibrative-type (hENT1, hENT2) and three concentrative-type transporters (hCNT1, hCNT2, hCNT3)^[17-19]^. Among these, hENT1 functions as the major gemcitabine transporter; *in vitro* experiments have demonstrated that increased expression of hENT1 is the critical factor for sensitivity to gemcitabine^[20]^. Restriction of intracellular uptake of gemcitabine by suppressed expression of hENT1 is one of the established mechanisms of drug resistance^[19,21]^. The majority of studies on patients with resected pancreatic cancer have suggested that high expression of this hENT1 may be predictive of improved survival in patients treated with gemcitabine^[22-24]^. Disrupted expression of hENT2 on the plasma membrane causes impaired uptake of gemcitabine, resulting in acquired chemoresistance of pancreatic cancer cells^[25]^.

Currently, several approaches to enhancing the efficacy of gemcitabine uptake or to bypass the hNTs have been introduced. hCNT1 is frequently diminished in pancreatic cancer cells compared with normal pancreatic ductal epithelial cells^[26]^, so drug inhibition or degradation of hCNT1 can increase the transportation of gemcitabine, and thus improve its efficacy^[27]^. A recent study indicated that mucin 4 (MUC4) suppresses hCNT1 expression and that inhibition of MUC4 enhances gemcitabine sensitivity^[28]^.

**Figure 3:**
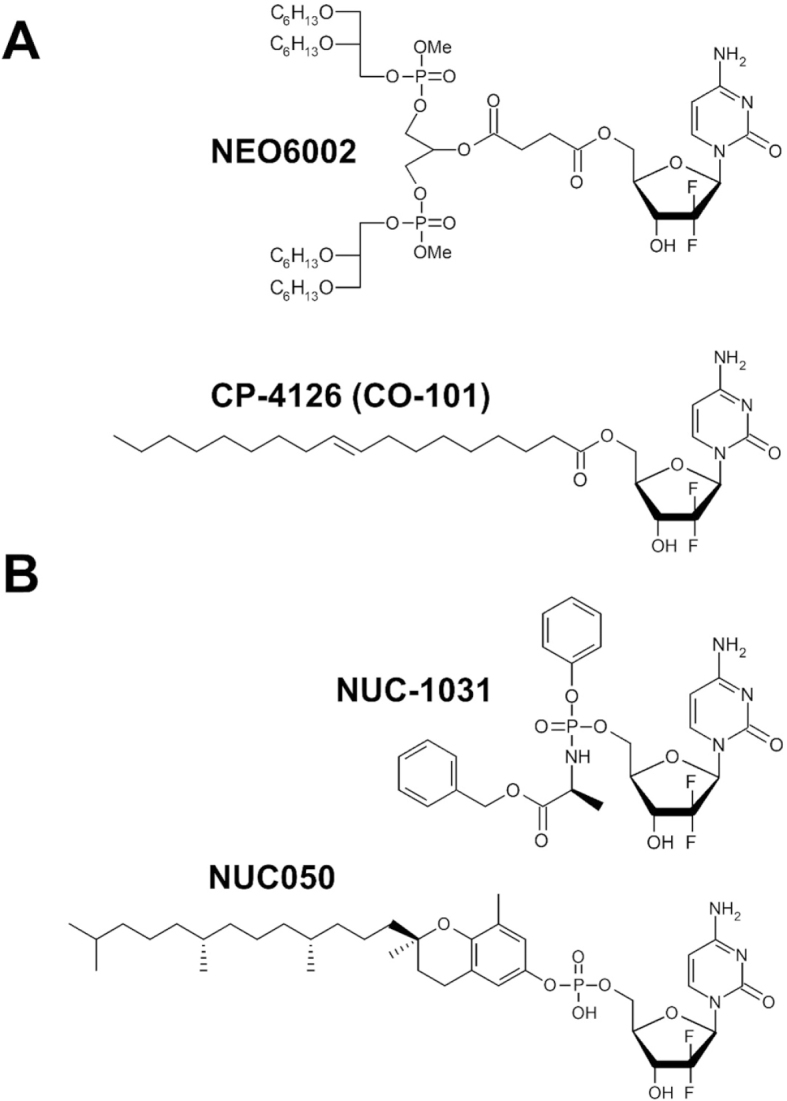
Structures of NEO6002 and CP4126, the gemcitabine-modified compounds that can bypass hNT-mediated introduction inside the cell (A); structures of NUC-1031 and NUC050, gemcitabine-modified compounds that can bypass the deoxycytidine kinase-mediated activation pathway of gemcitabine (B)

NEO6002 is a gemcitabine modified cardiolipin [Figure 3A]. This molecule entes the cell independently of hNT, and exerts higher activity, with lower toxic adverse side effects in mouse tumour xenograft model^[29]^. Another lipophilic prodrug, gemcitabine-elaidic acid conjugate CP-4126 [Figure 3A], also known as CO-101, is transported into the cells independently of hENT1 and has been demonstrated to be effective *in vitro* and in various human cancer models^[30]^. However, a long-term survival analysis found that the survival rate of patients using CP-4126 was not superior to gemcitabine in patients with low expression of hENT1 in metastatic PDAC (NCT01124786) [Table 1]^[31]^. This study was performed using an antibody against hENT (clone SP120), but recent report by Raffenne *et al*.^[32]^ using another antibody for hENT1 showed different results. They used a clone 10D7G2 and demonstrated that hENT tumour expression was significantly associated with better DFS and OS in PDAC patients. Thus, the usefulness of CP-4126 should be re-evaluated.

**Table 1:** Summary of clinical trials

Responsible party	Condition or disease	Intervention/treatment	Baseline participants (analyzed participants)	Age mean years (SD)	PFS median months (95%CI)	CR + PR % (95%CI^b^)	OS median months (95%CI)	NCT Number	Ref.
Clovis Oncology, Inc.	Metastatic Pancreatic Adenocarcinoma (hENT Low)	CO-101	182(114)	62.5 (9.62)	-	-	5.7 (4.7-7.6)	01124786	[31]
		Gemcitabine	185(118)	60.5 (11.19)	-	-	6.1 (5.2-7.7)		
National Cancer Institute (NCI)	Non-Small Cell Lung Cancer	FdCyd + THU	25(25)	59.92 (9.95)	2.3 (1.6-3.9)	0.0 (0.0-13.7)	N/A	00978250	*
	Breast Cancer		30(29)	56.04 (10.07)	3.7 (1.8-5.3)	6.9 (0.8-22.8)			
	Bladder Cancer		18(18)	67.77 (8.93)	3.6 (1.7-8.0)	5.6 (0.1-27.3)			
	Head and Neck Cancer		22(21)	55.17 (11.70)	1.7 (1.7-4.5)	0.0 (0.0-16.1)			
Imperial College London	Several types of cancers	NUC-1031	68(49)	56.3 (range 20-83)	4.0 (range 1-25)	10	N/A	01621854	[84]

*https://www.clinicaltrials.gov/ct2/show/results/NCT00978250. FdCyd: 5-Fluoro-2’-Deoxycytidine; THU: Tetrahydrouridine; PFS: progression-free survival; OS: overall survival; CR: complete response; PR: Partial response; CI: Confidence interval; SD: Standard deviation

Recently, nanoparticles loaded with gemcitabine have been developed. Nanoparticle encapsulation allows chemotherapeutic drugs to pass easily without being affected by cell surface NTs. GEM-HSA-NP is a gemcitabine-loaded albumin nanoparticle; using patient-derived xenograft models, this nanoparticle has been shown to be more effective than gemcitabine in inhibition of tumour growth, irrespective of expression levels of hENTs^[33]^. Squalenoyl-gemcitabine bioconjugate (SQdFdC) is self-assembled into a stable nanoparticle^[34]^. This particle passively diffuses into cancer cells, mainly accumulated within the cellular membrane including those of endoplasmic reticulum. Subsequently, it is released gradually into the cytoplasm and cleaved into dFdC^[35]^. This is an original transporter-independent pathway, and SQdFdC can overcome the acquired resistance in a transporter-deficient human leukemic cell line, *in vivo*^[35]^. Chitkara *et al*.^[36]^ made gemcitabine conjugated to poly (ethylene glycol)-block-poly (2-methyl-2-carboxyl-propylene carbonate) (PEG-PCC) which could self-assemble into micelles of 23.6 nm. These micelles were shown to afford protection to gemcitabine from plasma metabolism. Wonganan *et al*.^[37]^ created PLGA-b-PEG-OH nanoparticles incorporated with gemcitabine. They delivered gemcitabine effectively into hCNT-decreased tumour cells and were significantly more cytotoxic than free gemcitabine. These nanoparticles are summarized in Table 2.

**Table 2:** Examples of gemcitabine-containing nanoparticles and their effects

Nanoparticle	Ingredient	Outcome		Ref.
GEM-HSA-NP	albumin	*in vitro*	Inhibited cell proliferation, arrest cell cycle and induced apoptosis in pancreatic cancer cell lines.	[33]
		*in vivo*	More effective than gemcitabine when inhibiting tumour growth whether the expression levels of hENT1 were high or low in PDX models. The biotoxicity did not increase compared with gemcitabine.	
SQdFdC	squalene	*in vitro*	Exhibited superior anticancer activity in human cancer cells and gemcitabine-resistant murine leukaemia cells.	[34]
		*in vivo*	Exhibited superior anticancer activity in experimental leukemic mouse modes both after intravenous and oral administration.	
PEG-PCC GEM	PEG-PCC	*in vitro*	Induced cell apoptosis in pancreatic cancer cell lines	[36]
		*in vivo*	Significantly inhibited tumour growth in xenograft bearing mice	
PLGA-b-PEG-OH GEM	PLGA-b-PEG-OH	*in vitro*	Effectively delivered gemcitabine into hCNT-decreased ovarian cancer cells and showed significant cytotoxicity compared to free gemcitabine.	[37]

hENT1: human equilibrative nucleoside transporter 1; hCNT: human concentrative nucleoside transporters

The above mentioned strategies are promising delivery systems to address transporter-deficient resistant cancer in the clinical setting.

## Regulation of CDA expression and CDA inhibitors

Cytidine deaminase (CDA) is a ubiquitously expressed enzyme that catalyses cytidine and deoxycytidine into uridine and deoxyuridine, respectively. This enzyme participates in the pyrimidine salvage pathway that maintains the nucleotide pool balance for DNA and RNA synthesis. The great majority of gemcitabine is inactivated mainly by CDA [Figure 2], that mediates conversion from gemcitabine to difluorodeoxyuridine (dFdU)^[38]^. After deamination of gemcitabine, the metabolite is not further degraded but excreted from the cell^[39]^. CDA is activated in many organs, and dFdU is the major form of *in vivo* clearance which is the sole metabolite in the urine^[40]^. CDA is released from the cell and is found in the serum^[41]^; CDA has been detected in patients with several cancer types and correlates with responses to chemotherapy^[42,43]^. The *CDA* gene is affected by several genetic alterations, and marked variations in function ranging from null to increased activity have been observed^[44]^. A study conducted on pancreatic cancer patients with gemcitabine treatment demonstrated a correlation between CDA activity and chemoresistance and concluded that patients with 6U/mg or higher of CDA activity showed progression of disease by five-fold or more^[45]^. A recent systemic review concludes that *CDA* 79A > C polymorphism is a potential biomarker for toxicity of gemcitabine-based chemotherapy and that CDA testing is preferential before administration of gemcitabine^[46]^.

**Figure 4:**
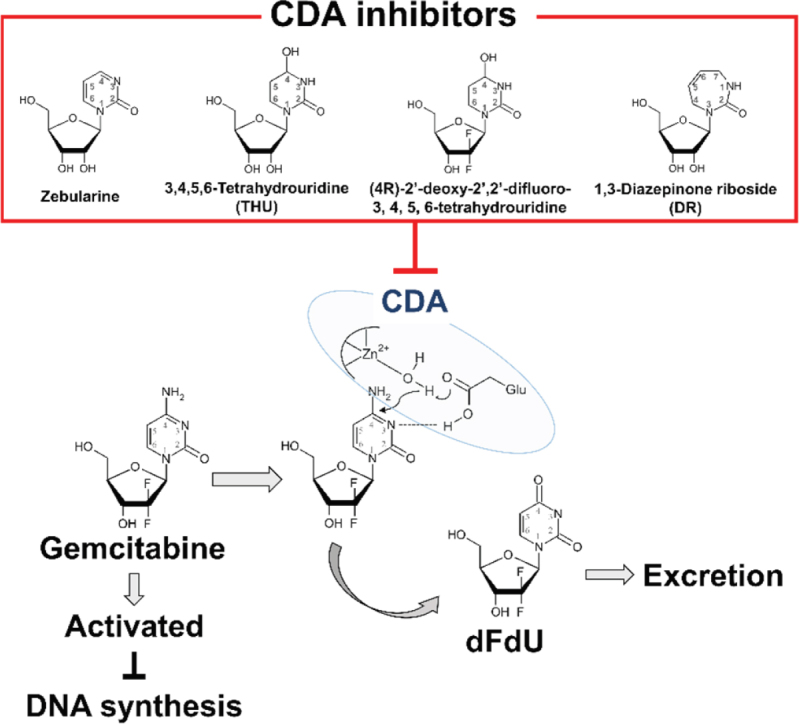
CDA-mediated processing of gemcitabine for excretion, and CDA inhibitors Zebularine, 3,4,5,6-Tetrahydrouridine (THU), 1,3-Diazepinone riboside (DR), and (4R)-2’-deoxy-2’,2’-difluoro-3 4, 5, 6-tetrahydrouridine. CDA: cytidine deaminase

CDA upregulation decreases the cellular gemcitabine concentration [Figure 4], and several studies have reported that increased CDA activity associates with gemcitabine resistance in cancer cells. A hematopoietic cell line with overexpression of CDA showed resistance to gemcitabine (2.4-fold in IC_50_ and 2.5-fold in IC_80_)^[47]^. On the other hand, studies using human tumour cell lines and tumour xenografts reported no association between chemoresistance and CDA activity^[48,49]^. These data showed that CDA is not the only determining factor for gemcitabine sensitivity *in vivo*, but its modulation may defeat chemoresistance.

In cancer cells, aberrations of the copy number of the *CDA* gene are not reported. *CDA* expression is mainly regulated transcriptionally and/or post-transcriptionally. *CDA* expression in most cancers is lower than in corresponding normal tissues because of DNA methylation in the promoter region^[50,51]^. miRNAs also regulate CDA expression; miR-484 directly inhibits CDA translation by targeting CDA 3’UTR and induces chemoresistance in breast cancer cells^[52]^, and decreased expression of miR-608 correlates with upregulation of CDA to induce chemoresistance in pancreatic cancer cells^[53]^. Albumin-conjugated paclitaxel (nab-paclitaxel) was shown to reduce the CDA protein by producing reactive oxygen species in a mouse pancreatic cancer model; this evidence may explain the usefulness of gemcitabine plus nab-paclitaxel (GnP)^[54]^.

Pharmacological inhibitors of CDA include zebularine, 3,4,5,6-tetrahydrouridine (THU) and 1, 3-diazepinone riboside (DR) [Figure 4]. Zebularine was first described in 1980^[55]^; it blocks CDA by way of a tetrahedral intermediate using the proton at C4 of pyrimidine ring^[56]^. However, zebularine also interacts with DNA methyltransferase; zebularine is not specific to CDA^[57]^. THU was first identified and purified in 1967 using an affinity capture method with CDA as bait^[58]^. The inhibitory action of THU is based on its C4 hydroxyl group in the pyrimidine ring. Since the bioavailability of THU is weak^[59]^, a new fluorinated version of this drug termed (4R)-2’-deoxy-2’,2’w-difluoro-3 4, 5, 6-tetrahydrouridine [Figure 4] has been developed with better oral bioavailability^[60]^. DR was discovered in 1981; it cannot interact with CDA through the water/zinc complex. Its inhibitory activity instead results from an electrostatic interaction utilizing π electrons of the DR ring and the benzene ring of the F137 of CDA, the catalytic site of the enzyme^[61]^. However, no results of DR effectiveness have yet been reported even in cultured cells.

As mentioned before, CDA high-expressing tumours are theoretically more resistant to cytidine-based therapies, including gemcitabine. With this assumption, several studies combining various chemotherapies and CDA inhibitors have been conducted to date. A Phase II clinical trial (ClinicalTraials.gov: NCT00978250, see Table 1), combining treatment with 5-fluoro-2’-deoxcytidine and THU, has just been completed; all 93 patients eligible for the study were assessed as PFS, including patients with advanced non-small cell lung cancer, breast cancer, bladder cancer, or head and neck cancer (https://www.clinicaltrials.gov/ct2/show/results/NCT00978250). Weizman *et al*.^[62]^ suggested that tumour infiltrating macrophages were responsible for stimulating the upregulation of CDA and acquisition of chemoresistance against gemcitabine in pancreatic cancer cells. Modulation of macrophage trafficking may offer a new strategy for response of cancer cells to gemcitabine^[62,63]^. Therefore, although CDA does not appear to be the only factor determining sensitivity to gemcitabine, its modulation remains a common strategy to overcome resistance.

## Transporters involved in efflux of gemcitabine and its metabolites

ATP-binding cassette (ABC) transporters are known to translocate a wide variety of substrates across the cell membrane and to mediate resistance against many therapeutic drugs, including anti-neoplastics and anti-infectives^[64]^. In addition, ABC transporters associate with a fraction of stem-like cells called side population (SP), refractory to Hoechst 33342 dye staining. This subpopulation was first isolated from murine hematopoietic cells^[65]^ and then from human cells. Isolated SP cells from various kinds of human solid cancers escape from chemotherapy due to overexpression of the ABC transporters^[66]^, and Borst reviewed pan-resistance and ABC transporters^[67]^.

Several studies examining the importance of ABC-transporters in gemcitabine resistance have confirmed that the abnormal expression of ABCB1, ABCC, and ABCG2 is associated with multidrug-resistance in pancreatic cancer^[68]^. On the other hand, MDR variants in two cell lines of small cell lung cancer showed increased DCK activity^[69]^, and human cancer cell lines overexpressing ABCB1 or ABCC1 showed increased sensitivity to gemcitabine^[70]^. Overexpression of ABCC4 and ABCC5 confer resistance to cytrabine and troxacitabine, but not gemcitabine^[71]^. Inhibition of one or even several ABCC transporters (ABCC3, ABCC5 and ABCC10) did not efficiently or completely inhibit efflux of gemcitabine^[72]^. Thus, the contribution of ABC transporters for gemcitabine resistance warrants further investigation.

## Prodrugs of DCK for bypassing the intracellular phosphorylation step

Once gemcitabine is transported into cells, phosphorylation by DCK is considered to be the major rate-limiting factor for activation. DCK has a Km value of 4.6 μmol/L for gemcitabine compared to 1.5 μmol/L for deoxycytidine, which makes this drug an appropriate substrate^[73]^. Gemcitabine is also phosphorylated by thymidine kinase 2. This is a mitochondrial enzyme which phosphorylates a broad range of natural nucleosides^[74]^, but its precise role for both gemcitabine host toxicity and anti-tumour activity is unclear^[7]^. Inactivation of DCK has been shown to be one of the key mechanisms for acquisition of gemcitabine resistance. The *DCK* gene is inactivated in all of the seven obtained gemcitabine-resistant cancer cell lines^[75,76]^. Knockdown of *DCK* leads to gemcitabine resistance in gemcitabine sensitive cell lines, while re-expression of *DCK* restored the chemo-sensitivity of gemcitabine in gemcitabine-resistant cell lines^[75,77,78]^. Clinical studies have shown that the DCK expression level in pancreatic cancer tissue is a reliable prognostic indicator of PFS, suggesting that DCK is a good biomarker of gemcitabine sensitivity for pancreatic cancer patients treated with gemcitabine^[79,80]^. Hu antigen R (HuR) is an RNA-binding protein that regulates DCK post-transcriptionally. HuR is strongly associated with the *DCK* mRNA level, and HuR-overexpressing cancer cells have been shown to be more sensitive to gemcitabine treatment^[81,82]^.

Modification of phosphorylated gemcitabine to bypass DCK-mediated activation may be an effective way to improve its function. NUC-1031 [Figure 3B] is a gemcitabine phosphoramidate prodrug that is produced by ProTide Technology^[83]^. NUC-1031 enters into the cell independently of the hENT1 transporter and does not require activation by DCK. Similar to the phosphorylated forms of gemcitabine, NUC-1031 is not subject to breakdown by CDA. In a Phase I study (NCT01621854), NUC-1031 demonstrated clinically significant anti-tumour activity even in patients with prior gemcitabine exposure and in cancers not traditionally perceived as gemcitabine-responsive [Table 1]^[84]^. A global randomized study (NuTide:121) including 828 patients with untreated advanced biliary tract cancer is ongoing^[85]^. NUC-1031 is the first anti-cancer drug with which ProTide has achieved initial success in clinical trials.

Δ-Tocopherol-monophosphate gemcitabine (NUC050) is a vitamin E phosphate nucleoside prodrug [Figure 3B] designed to bypass two mechanisms of gemcitabine resistance: downregulation of hNTs, and downregulation of DCK. Incorporation of NUC050 is not affected by hNTs, suggesting that it can bypass them. NUC050 retains most of the activity in DCK deficient cells, indicating that gemcitabine monophosphate is delivered in the cell^[86]^.

Further formulation development will increase the safety and efficacy of these prodrugs to overcome the cancer chemoresistance induced by the down-regulation of DCK.

## Radiation-induced activation of DCK

Most studies searching for synergism of radiation in combination with chemotherapeutic agents, including nucleoside analogues have been attempted to achieve radiosensitization of cancer cells. Gemcitabine is also employed clinically as a radiosensitizer^[87]^. The contribution of nucleoside analogues to synergic effects is thought to involve inhibition of DNA repair and modulation of nucleotide synthesis and availability. An alternative explanation for the synergism between radiation and nucleoside analogues is radiation-mediated chemosensitization. A number of studies have demonstrated that radiation alone can enhance the activity of DCK^[88-90]^. One previous study showed that DCK is phosphorylated at S74 by the DNA damage responsive kinase ATM, and may be activated^[91]^; this indicates a direct link between radiation and DCK activation. Another study showed that the ATM related kinase ATR is also involved in phosphorylation of DCK at S74^[92]^. S74Q mutation of DCK increases *K*_cat_ values by 11-fold for deoxycytidine and 3-fold for gemcitabine^[93]^. This in turn would explain the higher levels of active gemcitabine.

Recently, neoadjuvant therapy including radiation concurrent with gemcitabine has been conducted for borderline resectable pancreatic cancer^[8]^. Radiation may improve the cytotoxicity of gemcitabine by enhancing DCK activation.

## Conclusion

Gemcitabine-based chemotherapy remains a cornerstone of treatment for patients with advanced cancers. Chemoresistance against gemcitabine is multifaceted; therefore, pursuing the improvement of this chemotherapy is still an important challenge. Novel methodologies are required to improve patients’ prognoses.

In order to achieve an effective gemcitabine concentration within tumour cells, several considerations are needed. Nanoparticle-based medicine (nanomedicine) has numerous advantages compared with conventional medicines, including being able to protect gemcitabine from degradation, and provide a targeting delivery system. Some nanomedicines can accumulate inside tumour cells by the incorporation of ligands that target molecules overexpressed on the cancer cell surface^[94]^. Elechalawar *et al.*^[95]^ developed a targeted drug delivery system to pancreatic cancer using gold nanoparticles as the delivery vehicle, the anti-EGFR antibody cetuximab (C225/C) as the targeting agent, gemcitabine as the effective drug, and polyethylene glycol (PEG) as the stealth molecule. This nanoconjugate, termed ACG44P1000, showed enhanced cellular uptake and cytotoxicity to pancreatic cancer cell lines *in vitro* study. Although the effect of this nanoconjugate may be limited, further investigations will lead to more effective improvements.

Tumours are heterogeneous and exhibit molecular complexity, with significant variation among patients. Treatments of cancer patients require precision medicine-based genetic and biomolecular characteristics. The traditional chemotherapeutic approach (one-size-fits-all) can lead to unnecessary exposure to adverse side effects without the anticipated survival benefits^[96]^. In the last decade, improvements in high-throughput sequencing methods and profiling of transcripts have led to the discovery of many new targets for treatments. The identification of receptor overexpression in cancer cells will lead to the development of nanomedicines to improve the selectivity to the cancer cells and reduce off-target toxicities of gemcitabine. Further studies are needed for gemcitabine-based treatment to be included in personalized medicine tailored for numerous molecular therapeutic targets in multiple pathogenic pathways.

## Declarations

### Copyright

© The Author(s) 2020.
